# Different Workplace Currencies and Employee Voice: From the Multidimensional Approach of Leader–Member Exchange

**DOI:** 10.3389/fpsyg.2020.00589

**Published:** 2020-04-28

**Authors:** Qiwei Zhou, Da Huo, Fan Wu

**Affiliations:** ^1^School of Economics and Management, Beijing University of Chemical Technology, Beijing, China; ^2^School of Economics and Management, Dalian University of Technology, Dalian, China; ^3^School of Economics and Management, Tsinghua University, Beijing, China

**Keywords:** leader–member exchange, social currency, work-related currency, promotive voice, prohibitive voice

## Abstract

Building upon social exchange theory and the current voice research, we posit that employee workplace “currencies of exchange” with the leader (i.e., social currency and work-related currency) are key predictors of employee promotive and prohibitive voice. Furthermore, we distinguish between the different roles of social currency and work-related currency in predicting promotive and prohibitive voice, respectively. More importantly, this study further explores the moderating effects of two important individual characteristics, psychological safety and power distance orientation, on the relationships between currencies and voice. We randomly sampled 598 Chinese employees via an online survey platform to test our hypotheses. Our results show that both social currency and work-related currency are determinants of promotive voice and prohibitive voice. Moreover, the boundary conditions for the two kinds of currencies are different. Specifically, employee psychological safety strengthens the influence of social currency on both types of employee voice, while employee power distance orientation could only amplify the relationship between work-related currency and promotive voice. Our research provides important implications for both theory and practice. Limitations and future directions are also discussed.

## Introduction

Great changes have taken place in the business world during recent years. In order to survive in the progressively competitive environment, organizations have to be increasingly adaptive to changes. This requires proactive behaviors and contributions from every employee in the organization (Fuller et al., 2006). To this point, Morrison (2011) summarized the importance of voice and noted that the extent to which employees are willing to express their concerns and offer their suggestions about key issues can have a critical impact on operating performance and organizational survival. In view of the increasingly uncertain and complex business environment in recent years, voice has drawn numerous scholarly attention (e.g., Burris et al., 2013; Bashshur and Oc, 2015; Hilverda et al., 2018; Qian et al., 2018; Gao and Jiang, 2019).

Voice emphasizes the expression of constructive challenge for the benefit of organizations (Van Dyne and LePine, 1998). Employees communicate ideas and opinions about work-related issues. They make suggestions that are intended to improve organizational performance (Van Dyne et al., 2003; Morrison, 2011). Because of differences in the contents, Liang et al. (2012) proposed that there are two types of voice: promotive voice and prohibitive voice. Promotive voice refers to the expressions of new ideas and suggestions to improve efficiency (Liang et al., 2012, p. 71). Prohibitive voice is mainly related to the communication about existing problems which are harmful to the organization (Liang et al., 2012, p. 72). While this conceptual approach emphasizes different facets of voice, scholars suggest that research in this vein should continue to explore the antecedents of promotive voice and prohibitive voice in a more fine-grained manner (Liang et al., 2012; Morrison, 2014).

Furthermore, most existing studies focus on the different dispositional predictors of the two different types of voice (Lin and Johnson, 2015; Kakkar et al., 2016; Chamberlin et al., 2017; Huang et al., 2018). However, voice is one behavior that is sensitive to situational factors which tie closely with social interactions across different contexts (Kakkar et al., 2016). Individuals develop different patterns of interactions with others at the workplace (Ferris et al., 2009; LePine et al., 2012). Their relationships with the leaders can have a critical impact on voice behavior because the leader is one of the most important channels of speaking up (Withey and Cooper, 1989; Detert and Burris, 2007). Therefore, we draw from social exchange theory and aim to offer more explanation as to how and when individuals engage in promotive and prohibitive voice. Specifically, we focus on the different roles of employees’ social and work relationship with leaders in predicting the different kinds of voice behavior.

In light of the development in the leader–member exchange (LMX) literature (Dienesch and Liden, 1986; Graen and Uhl-Bien, 1995), it is worth noting that scholars started to explore the predictors of voice through the lens of LMX (e.g., Bhal and Ansari, 2007; Burris et al., 2008; Van Dyne et al., 2008; Botero and Van Dyne, 2009). Most prior studies suggest that better-quality relationships, in general, promote voice (e.g., Botero and Van Dyne, 2009; Morrison, 2011). This is because employees feel more comfortable to offer suggestions when they have good relationships with the leader (Stamper et al., 2009). As this research progresses, however, scholars started to challenge the notion that subordinates are always more willing to speak up when a high LMX is in place (Burris, 2012; Bernerth et al., 2016). In their recent review, Carnevale et al. (2017) noted that the underlying mechanism between LMX and voice is more complex. In fact, some have reported a curvilinear relationship between LMX and voice (Carnevale et al., 2019), and some even argued that a good relationship with the leader may hinder a subordinate’s desire to tell the truth in order to maintain a harmonious relationship with the leader (Morrison and Milliken, 2000; Milliken et al., 2003; Burris, 2012). In spite of these mixed findings in existing research, viewing LMX as a unidimensional construct does not allow us to explore the roles of distinct contents of exchange in explaining different kinds of employee voice behavior. In other words, little is known about the different effects of various aspects of exchange on promotive voice vs. prohibitive voice.

In order to uncover the complex relationship between LMX and voice, we adopt the multidimensional approach of LMX (Liden and Maslyn, 1998; Maslyn and Uhl-Bien, 2001). Specifically, Liden and Maslyn (1998) proposed that LMX development involves different “currencies of exchange” – affect, loyalty, professional respect, and contribution – and they developed the multidimensional measure of LMX (labeled LMX-MDM). By using these dimensions, Maslyn and Uhl-Bien (2001) further proposed that affect, loyalty, and professional respect can be categorized as social currencies, while contribution refers to work-related currency. These two kinds of currencies are related to different aspects of exchange relationships. Whereas work-related currency stands for the interactions on job-related issues, social currency denotes non-job-related interactions (Bhal and Ansari, 2007). Not only do social and work-related currencies represent different dimensions of LMX; they may also lead to different behaviors and outcomes (Bhal and Ansari, 2007). Integrating research on dimensionality of LMX and voice can therefore allow us to further unpack the distinct influences of different kinds of interactions embedded in LMX on employee promotive and prohibitive voice.

Furthermore, we aim to further contribute to this research by exploring the boundary conditions of social currency and work-related currency in predicting promotive and prohibitive voice, respectively. We focus on two important individual characteristics: psychological safety and power distance orientation, and we examine the extent to which these individual factors may affect employee voice behavior in conjunction with relational predictors. This is because the extent to which individuals are willing to share their opinions or express their concerns about existing issues is contingent upon their perceived risks of being punished or misunderstood (Detert and Burris, 2007; Liang et al., 2012) and their sensitivity to changes in the *status quo* and leader behavior (Eylon and Au, 1999; Daniels and Greguras, 2014). The exploration of these moderators can help us better understand the interactive effects of both relational factors and individual factors on voice.

**FIGURE 1:**
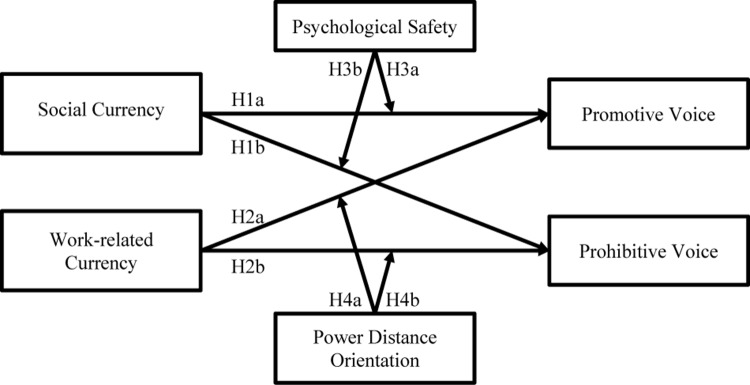
The theoretical model of our research.

In summary, we hope to advance the understanding of how and when employees engage in promotive and prohibitive voice by taking the multidimensional LMX perspective and exploring the influences of social and work-related currencies on the two kinds of voice. We also posit that the effects of social and work-related currencies on promotive and prohibitive voice are contingent upon the consideration of psychological safety and power distance orientation. In doing so, we contribute to both LMX literature and voice research by shedding new light on the underlying mechanisms regarding how various aspects of employees’ relationships with leaders affect their voice behavior in separate ways while taking into account their dispositional characteristics. To address these issues, we conducted an online survey and randomly sampled 598 Chinese employees from a registered participant pool. Figure 1 depicts our theoretical framework.

This paper is organized as follows. First, we review the literature on promotive and prohibitive voice and develop theoretical arguments that predict these two kinds of voice through the multidimensional LMX perspective. Second, we develop hypotheses regarding how psychological safety and power distance orientation moderate these relationships. Next, we describe our research design and empirical results. We then discuss the theoretical contributions and practical implications of this study. Limitations and promising future research directions are also addressed.

## Theory and Hypotheses

### Voice as Both Promotive and Prohibitive

Voice is the “discretionary communication of ideas, suggestions, concerns, or opinions about work-related issues with the intent to improve organizational or unit functioning” (Morrison, 2011, p. 375). However, studies have also considered the possibility that voice can simultaneously be a self-serving behavior that helps employees promote a positive self-image in front of others (Burris, 2012; Klaas et al., 2012; Morrison, 2014; Weiss and Morrison, 2019). Despite these different motives, voice has mostly been viewed as favorable to the workplace at different levels. At the firm level, voice can lead to an improved decision-making process (Morrison and Milliken, 2000) and better organizational performance (Gittell et al., 2004; Bryson et al., 2013). At the team level, voice is positively related to team innovation (Dreu, 2002) and performance (Dooley and Fryxell, 1999). At the individual level, research shows that voice can lead to higher affective commitment (Thomas et al., 2010), better physical and mental health (Cortina and Magley, 2003; Morrison, 2011), and lower voluntary turnover (Spencer, 1986). Given the benefits of voice at different levels, scholars have devoted numerous attention in exploring the various factors that promote employee voice behavior (e.g., Chamberlin et al., 2017; Hilverda et al., 2018; Qian et al., 2018; Gao and Jiang, 2019). Within this research, one stream focuses on individual characteristics such as dispositional factors and attitudinal factors in predicting voice (e.g., Janssen et al., 1998; LePine and Van Dyne, 1998, 2001; Nikolaou et al., 2008). Meanwhile, another stream examines the impact of situational factors on employee voice behavior. As Dutton et al. (2002) suggested, employees search for cues from their surroundings in making a decision whether to speak up or to stay silent. Specifically, research has explored factors such as organizational structure (Glauser, 1984), culture (Dutton et al., 1997, 2002), team size (LePine and Van Dyne, 1998; Islam and Zyphur, 2005), and team climate (Zhou and George, 2001) as social cues that could influence employee voice behavior.

In another vein, Liang et al. (2012) offered a more fine-grained content-based perspective for exploring voice behavior. Following their categorization, recent work has shown that promotive and prohibitive voice have different antecedents. For example, Kakkar et al. (2016) examined the relationship between different dispositional factors – approach orientation and avoidance orientation – and the two kinds of voice. They found that approach orientation and avoidance orientation affect promotive voice and prohibitive voice in opposite ways. In addition, Chamberlin et al. (2017) explored various factors that would influence promotive and prohibitive voice. They showed that factors such as core self-evaluation, felt responsibility, organizational commitment, psychological safety, ethical leadership, and leader openness are more strongly related to promotive voice, while detachment, behavioral inhibition, and performance-avoidance goal orientation are more associated with prohibitive voice. These studies, however, mostly focused on the different dispositional factors in explaining promotive and prohibitive voice. There have been few studies that examined how relational factors may lead to promotive and prohibitive voice in different ways. This is glaring because employees are likely to engage in different kinds of voice behavior across different contexts of social interactions. In this study, we explore the role of these different predictors of promotive and prohibitive voice by drawing from the multidimensional perspective within the LMX literature.

### Currencies of Exchange and Voice

Leader–member exchange theory (Graen and Uhl-Bien, 1995) holds that leaders develop unique relationships with each of their subordinates. Due to time constraints, leaders selectively develop close relationships with a few subordinates (Dienesch and Liden, 1986). With the other subordinates, leaders rely more on formal rules and authority. In another words, there are two types of LMX: the “in-group” and the “out-group” exchange. In essence, the in-group subordinates have more opportunities to interact with their leaders. They receive more trust, support, and rewards. The out-group members have fewer chances to interact with leaders, and they receive less support and rewards (Dienesch and Liden, 1986). As a result, some employees will be less willing to spend time on extra-role behaviors than others (Liden and Graen, 1980).

The LMX literature is grounded within social exchange theory (Gouldner, 1960; Blau, 1964), which states that people exchange numerous materials such as information and advice as they develop friendship with colleagues at the workplace (Krackhardt, 1990; Brass and Burkhardt, 1992; Sparrowe and Liden, 1997). On the basis of this social exchange, scholars argue that LMX is multidimensional such that the contents of exchange between leaders and members vary along different dimensions (Dienesch and Liden, 1986; Liden and Maslyn, 1998). Specifically, Liden and Maslyn (1998) proposed that LMX development involves different currencies of exchange – affect, loyalty, professional respect, and contribution – and they developed LMX-MDM. Building on these dimensions, Maslyn and Uhl-Bien (2001) further proposed that affect, loyalty, and professional respect can be seen as social currencies, while contribution denotes work-related currency.

Although both social and work-related currencies are important to the development of LMX, they may exert different impacts on individual behaviors and dyadic outcomes because the different types of exchange are likely to influence an employee’s behavior in distinct ways (Dienesch and Liden, 1986; Maslyn and Uhl-Bien, 2001). This notion is aligned with the theoretical underpinning of the LMX research that delineates the importance of the situational context in which individuals interact (Biddle, 1986; Johns, 2006; Yu et al., 2018) and the nature of these interactions in affecting individuals’ behaviors (Graen and Uhl-Bien, 1995; Erdogan and Liden, 2002). However, the body of research that examines the impact of LMX on voice has mostly viewed LMX as the overall quality of the relationship between leader and subordinates (e.g., Van Dyne et al., 2008; Botero and Van Dyne, 2009; Morrison, 2011; Liu et al., 2013). Few studies have examined the differential impacts of the different kinds of exchange relationships on voice. This makes it more difficult to ascertain the roles of social currency as opposed to work-related currency in predicting promotive voice, and prohibitive voice, or vice versa. In order to address this gap, we build on the multidimensional perspective within the LMX literature and theorize the mechanisms through which the two kinds of voice are influenced by social currency and work-related currency.

#### Social Currency and Voice

From the multidimensional perspective of LMX, social currencies refer to the social components of the exchange relationship between the leader and the employee. In general, more social currencies indicate a better-quality social relationship with the leader. Social currencies include three dimensions of LMX – affect, loyalty, and professional respect (Maslyn and Uhl-Bien, 2001). In spite of the different underlying mechanisms, we posit that social currencies are positively related to both promotive voice and prohibitive voice.

In terms of promotive voice, employees who have accumulated more social currencies with their leaders are more likely to make extra efforts in searching for ways that could improve existing practices and help the organization. To this point, scholars noted that employees are more likely to engage in prosocial behaviors when they have better-quality social relationships with their bosses (Stamper et al., 2009). This can be explained by the positive associations with voice among the three dimensions of social currencies. Stated differently, we argue that social currencies in the form of affect, loyalty, and professional respect are positively related to promotive voice.

First, the affective dimension of LMX stands for friendship and liking that the dyadic members feel toward each other (Liden and Maslyn, 1998; Maslyn and Uhl-Bien, 2001). Voice behavior can be seen as an affective response to the mutual relationship with the leader such that employees are more willing to spend time and effort to search for new ideas that could benefit the leader and the organization (Spector and Fox, 2002; Ilies et al., 2006). Similarly, the loyalty aspect of LMX can also encourage employees to find ways that could improve the overall functioning of their work unit in order to better support their leader (Maslyn and Uhl-Bien, 2001). Third, professional respect describes the perception of professional capabilities between the dyads (Liden and Maslyn, 1998; Maslyn and Uhl-Bien, 2001). A higher level of professional respect, in turn, holds the employee to a higher standard in order to maintain such a high level of respect. It demands that such an employee continuously put in extra effort to keep finding better ideas or solutions than other colleagues. Taking these together, we posit that:

***H1a: Social currency is positively related to employee promotive voice.***

In terms of prohibitive voice, we argue that employees with more social currencies, in spite of the different manifestations along the three dimensions, are more likely to express concerns about harmful issues. Engaging in prohibitive voice behavior typically involves more personal risks than does promotive voice. This is because expressing concerns about existing problems or harmful practices would indicate dysfunction or deficiencies of current leadership (Liang et al., 2012). The perception of risks and the fear of facing negative social consequences generally hinder employees’ willingness to engage in prohibitive voice. Nonetheless, social currencies, in the form of affect, loyalty, and professional respect, can help relieve this sense of personal risks and thus promote prohibitive voice.

Specifically, employees with affective leader–member relationships usually have more chances to communicate with leaders in non-work settings (Law et al., 2000). With these additional opportunities to communicate with their leaders, employees learn to better understand the preferences and intentions of their leaders. As such, their perception of potential risks and fear of being misunderstood from engaging in prohibitive voice can be reduced.

Loyalty reflects the extent to which members and leaders publicly support and defend each other’s actions and character (Dienesch and Liden, 1986; Liden and Maslyn, 1998). An employee who possesses a higher level of loyalty toward his or her supervisor generally feels safer, compared to those who do not, taking on risky endeavors such as voicing concerns or pointing out key issues. As a result, this perception of absence of negative consequences can motivate employees to engage in prohibitive voice (Liang et al., 2012).

In addition, we further contend that professional respect can also lead to a reduced sense of personal risks or fear about negative consequences associated with expressing concerns. Professional respect arises when each member of the leader–follower dyad has developed a reputation about his or her capabilities and professionalism (Liden and Maslyn, 1998). This, in turn, makes the employee more comfortable with expressing concerns, as he or she believes that the leader can understand and respect his or her behavior. Taking these together, we posit that:

***H1b: Social currency is positively related to employee prohibitive voice.***

#### Work-Related Currency and Voice

Work-related currency refers to the contribution dimension of LMX. In particular, contribution is reflected in the perception between dyadic members regarding the extent to which the other party completes the tasks within and beyond the job description (Liden and Maslyn, 1998). Such a perception is developed as the dyadic members perform work-related activities and grows over time as the exchange relationship evolves. Whereas social currency emphasizes the interpersonal aspect of the exchange relationship, the core premise of work currency is the completion of task-oriented activities (Liden and Maslyn, 1998). Voice behavior, in the form of expression of new ideas and creative solutions, can be seen as the result of the growing exchange relationship around assignment and completion of different tasks. This is because both parties of the exchange relationship with a high level of work-related currency put forth more energy and resources in order to accomplish mutual goals at work. As a result, they are more prone to embrace new ideas or solutions that could benefit the organization.

Furthermore, an exchange relationship with the leader can influence regulatory foci of the employees (Brockner and Higgins, 2001). Specifically, employees with more work-related currencies will be more promotive focused since both the leaders and the followers can benefit from the development of the work unit. In turn, they pay more attention to the positive things at work and remain open to changes (Kark and Van Dijk, 2007). These promotive-focused employees are therefore more likely to offer additional insights or new ideas that could lead to further improvement of the work unit or the organization. In fact, research has shown a positive link between employees’ promotion focus and their promotive voice behavior (Lin and Johnson, 2015).

In addition, promotive voice behavior can be seen as a way through which an employee tries to obtain or maintain a strong impression among peers by making a greater contribution at work. Research on impression management has noted that individuals are likely to engage in certain behaviors in order to better manage others’ impressions of themselves (Wayne and Liden, 1995). On the one hand, making more contributions at work by offering creative ideas or innovative solutions can lead to an improved impression among peers. Motivated by their desire to obtain a strong impression, employees are more likely to engage in promotive voice. On the other hand, an employee with more work-related currencies can be held to a higher standard, as he or she is expected to make continuous contributions at work. This requires the employee to keep finding new ideas and better solutions in order to maintain his or her colleagues’ impression of him or her. Synthesizing extant theorizing, we argue that work-related currency manifested in the contribution dimension of LMX is positively related to voice. Thus, we propose:

***H2a: Work-related currency is positively related to employee promotive voice.***

We theorized earlier that more work-related currency can make employees more willing to spend time and energy on finding ways to improve their workplace. Therefore, a higher level of work-related currency can lead to more promotive voice, such as making suggestions or introducing new ideas. However, having more work-related currency between the dyads will lead to less prohibitive voice. In other words, we argue that a higher level of work-related currency is negatively related to prohibitive voice of employees.

Specifically, a high level of work-related currency denotes recognition for one’s work that has been accepted by his or her leaders and peers (Maslyn and Uhl-Bien, 2001). On the basis of prior accomplishments, such an employee has developed an impression that he or she is capable of making a substantial contribution to the workplace. Engaging in prohibitive voice, however, can potentially distort this strong impression that he or she has managed to obtain. This is because expression of concerns about current practices can indicate inadequacy of work that an employee was part of, such that it undermines his or her prior contribution. Nevertheless, a high level of work-related currency demands that an employee make contributions on a continuous basis. Thus, an employee with a high level of work-related currency may try to avoid things that can create conflicting signals in order to maintain his or her impression among others.

Furthermore, engaging in prohibitive voice typically incurs a high level of personal risks. Specifically, pointing out existing problems may indicate incompetency of current leadership or confront powerful others at work who are more comfortable with the *status quo* (Liang et al., 2012). As such, challenging existing practices can lead to a higher level of difficulty in making a future contribution to the workplace. Employees with more work-related currencies are likely to be more concerned about these additional obstacles, which can incur a greater level of stress. Taking these together, we posit that work-related currency manifested in the contribution dimension of LMX is negatively related to prohibitive voice.

***H2b: Work-related currency is negatively related to employee prohibitive voice.***

### Moderating Role of Psychological Safety

Many employees would keep silent rather than speak up (Milliken et al., 2003). They are reluctant to express their concerns about problems of the organizations to their leaders. Social information processing theory (Salancik and Pfeffer, 1978) suggests that employees’ attitudes and behaviors are influenced by contextual factors. Employees scan their workplace and develop a perception of their surrounding environment. Based on their perception, employees arrive at the decision whether to engage in certain behaviors such as voice. Voice behavior often entails risk, since offering constructive suggestions implies a challenge to the *status quo* (Liu et al., 2010). Such a behavior may damage the public image of the employee, may worsen interpersonal relationships (Dutton et al., 1997; Milliken et al., 2003), and can be subject to formal or informal sanctions (Pinder and Harlos, 2001). Thus, whether it is safe to voice would be the first consideration for an employee to speak up (Liang et al., 2012). Research has shown that psychological safety can promote expression of opinions among employees (Edmondson, 2003) and is positively related to voice behavior (Detert and Burris, 2007; Walumbwa and Schaubroeck, 2009; Liang et al., 2012; Tangirala et al., 2013; Liu et al., 2017). In contrast, when an employee feels that expressing opinions can cause trouble, he or she will try to avoid expression of his or her true opinions and remain silent.

Psychological safety refers to “being able to show and employ one’s self without fear of negative consequences of self-image, status or career” (Kahn, 1990, p. 708). The degree to which a subordinate feels psychologically safe is closely related to his or her quality of social relationships with the leader (Carmeli et al., 2009). Kahn (1990) proposes that better interpersonal relationships that offer support, trust, openness, and flexibility are typically associated with higher psychological safety. The mutual respect and interpersonal trust fostered by leaders would make employees have greater confidence in their relationships with leaders (Chen et al., 2019), which in turn increases the probability that the employees will speak up (Ajzen, 1991; Schaubroeck et al., 2011).

Nonetheless, social currencies include different contents, such as mutual liking, loyalty, and respect for the professional skills of supervisors. On the basis of the different constellations of social currencies, employees are willing to offer suggestions or to express concerns only when they feel safe speaking up. Thus, psychological safety serves as an important boundary condition in predicting voice. When good leader–member social relationships exist, higher psychological safety makes an employee feeling safer sharing opinions freely for the benefit of the organization. Such an employee will be less concerned about negative social consequences associated with his or her voice behavior. In other words, an employee with more social currency with the leader will be even more willing to speak up when a high level of psychological safety is in place. Conversely, an employee will be less willing to engage in voice behavior in spite of good social relationships with the leader if the employee feels psychologically unsafe speaking up. Stated differently, the positive influence of social currency on voice behavior will be weakened, since the employee may stay silent to keep harmony with the leader. In this vein, we hypothesize that employee psychological safety strengthens the relationship between social currency and promotive and prohibitive voice, respectively. Thus, we propose:

***H3a: Employee psychological safety moderates the relationship between social currency and promotive voice, such that the relationship is stronger when employee psychological safety is high rather than low.***

***H3b: Employee psychological safety moderates the relationship between social currency and prohibitive voice, such that the relationship is stronger when employee psychological safety is high rather than low.***

### Moderating Role of Power Distance Orientation

Our earlier theorizing suggests that employees’ work relationships with their leaders affect their voice behavior. We further propose that the impact of work relationship on voice is also influenced by cultural value–related differences held by different individuals. Studies have shown that an employee’s perceptions and responses to leader behavior can be influenced by different cultures or values (Kirkman et al., 2009; Brown and Mitchell, 2010; Lian et al., 2012). In recent years, scholars have begun to place greater emphasis on cultural value differences at the individual level (e.g., Farh et al., 1997; Kirkman and Shapiro, 2001; Farh et al., 2007; for a review, see Taras et al., 2010). To this point, scholars noted that this individual focus on cultural value differences can better capture the individual variability of value orientations within a culture (Farh et al., 2007; Botero and Van Dyne, 2009).

Among individual values, power distance orientation is arguably the most important to exchange relationships at the workplace (Chen and Aryee, 2007; Kirkman et al., 2009), especially between leaders and subordinates (Lin et al., 2013, 2018). It is the most relevant to our research framework because power distance orientation may directly influence the development of subordinates’ perception and their reaction to leaders through ongoing exchange (Kirkman et al., 2009; Hu et al., 2018). Furthermore, we focus on the role of power distance orientation in the relationship between work-related currency and voice because the influence of power distance largely unfolds in the work relationship between leaders and subordinates through their task-oriented interactions. In fact, Daniels and Greguras (2014) noted that high power distance is more task oriented. As we stated earlier, unlike how social currency captures the interpersonal aspect of the exchange relationship, work-related currency centers around assignment and completion of different tasks between leader and subordinates. Therefore, we propose that employee power distance orientation serves as an important moderator of the relationship between work-related currency and employee voice.

Power distance orientation can be defined as the extent to which an individual accepts the unequal distribution of power in an organization (Clugston et al., 2000; Farh et al., 2007). Employees with high power distance orientation tend to perceive that the existing of a power difference between the leader–employee dyad is legitimate (Kirkman et al., 2009). They are more sensitive to the changes in leader behavior and respond to the changes actively (Eylon and Au, 1999). As for employees with low power distance orientation, changes of leader behavior are less prioritized in guiding their behavior (Schaubroeck et al., 2007). Stated differently, employees with high power distance orientation defer more to the leader (Schaubroeck et al., 2007), and they are more prone to define their relationships with the leaders as work relationships.

In terms of voice behavior, the extent to which individuals are willing to share their opinions rests upon their attentiveness to changes in the *status quo* and the leaders’ behaviors (Eylon and Au, 1999). When employees realize that they have high-quality work relationship with the leader, they will have stronger psychological reciprocity (Francis, 2012) due to greater respect for the leader (Schaubroeck et al., 2007) and try to find ways to make contributions at work. Thus, they are more motivated and more likely to offer new ideas or creative solutions that may lead to improvement of their workplace. In other words, we argue that a high-quality interaction between a leader and employees at work can lead to more promotive voice behavior. As such, employee power distance orientation moderates the relationship between work-related currency and promotive voice such that the positive relationship between work-related currency and promotive voice is stronger when power distance orientation is high rather than low.

Furthermore, we posit that employee power distance orientation also moderates the relationship between work-related currency and prohibitive voice. We theorized earlier that a high-level work-related currency hinders an employee’s willingness to engage in prohibitive voice due to a higher level of perceived risk and greater concern about impression management. We further contend that such a relationship is even more negative when power distance orientation is high rather low. Specifically, employees with high power distance orientation are more likely to accept the *status quo* and less willing to challenge with the authority (Schaubroeck et al., 2007). In other words, they are more likely to be concerned about their leaders’ impression of them. Moreover, employees with higher power distance orientation typically have less demand for autonomy and prefer clear instruction at work (Alves et al., 2006). Engaging in prohibitive voice can cause a greater deal of stress for these employees. As a result, their willingness to engage in prohibitive voice, rather than following existing rules and authority, is further reduced by their high power distance orientation. Taking these together, we propose:

***H4a: Employee power distance orientation moderates the relationship between work-related currency and promotive voice, such that the relationship is more positive when employee power distance orientation is high rather than low.***

***H4b: Employee power distance orientation moderates the relationship between work-related currency and prohibitive voice, such that the relationship is more negative when employee power distance orientation is high rather than low.***

## Materials and Methods

### Participants and Procedure

We recruited participants via wjx.cn, a reliable Chinese online platform for data collection similar to Qualtrics Online Sample, and randomly distributed questionnaire links in the participant pool. In order to meet our requirements, participants had to be currently employed. During the 1-week data collection window, 702 participants answered our survey. Voluntariness and confidentiality were guaranteed to every participant before filling in their responses. This randomized distributing and recruiting process enabled us to cover a relatively diverse sample of individuals from different sectors with different backgrounds. After excluding cases with missing data or invalid responses (e.g., too-short answering time or same answers for each item), we retained a final sample of 598 participants. The valid response rate is 85.2%. This study was carried out in accordance with the recommendations of the ethics committee of Tsinghua University with written informed consent from all subjects. All subjects gave written informed consent in accordance with the Declaration of Helsinki. The protocol was approved by the ethics committee of Tsinghua University. Each participant received a small reward after completing the survey.

Among all participants, 51.2% were females, and 95.1% received at least a vocational/junior college degree. As for age, 11.5% were between 21 and 25 years old, 38.6% were between 26 and 30 years old, 27.3% were between 31 and 35 years old, 10.7% were between 36 and 40 years old, and 8.4% were between 41 and 45 years old. In terms of organizational tenure, 25.4% of participants had been working in the same company for 2–3 years, 22.2% were tenured between 4 and 5 years, and 17.6% had a tenure between 6 and 7 years.

### Measures

All survey items were in Chinese. In order to ensure accuracy, we followed Brislin’s (1986) recommendation of translation and back-translation procedures. Survey items were then finalized.

#### Currencies

Maslyn and Uhl-Bien (2001) proposed that the three dimensions of affect, loyalty, and professional respect in LMX are “social currencies” that focus on social exchange between leader and member, whereas the contribution dimension in LMX denotes “work-related currency” (Bhal and Ansari, 1996; Liden and Maslyn, 1998). We adopted LMX-MDM (Liden and Maslyn, 1998) for these two kinds of currencies.

Specifically, employees assessed their social currencies with nine items developed by Liden and Maslyn (1998). Sample items included “My supervisor is the kind of person one would like to have as a friend” [affect, 1 = *strongly disagree* to 7 = *strongly agree*; Cronbach’s alpha (*α*) = 0.87]; “My supervisor defends my work actions to a superior, even without complete knowledge of the issue in question” (loyalty, 1 = *strongly disagree* to 7 = *strongly agree*; *α* = 0.78); and “I respect my supervisor’s knowledge of and competence on the job” (professional respect, 1 = *strongly disagree* to 7 = *strongly agree*; *α* = 0.88). Cronbach’s alpha for this construct was 0.91.

Employees assessed their work-related currencies with two items representing the dimension of contribution in the LMX scale (Liden and Maslyn, 1998). Sample items included “I am willing to apply extra efforts, beyond those normally required, to further the interests of my work group” (1 = *strongly disagree* to 7 = *strongly agree*; *α* = 0.80).

#### Psychological Safety

Employees rated their psychological safety with a four-item measure adopted from Liang et al. (2012) developed within the context of China. A sample item included “I can express my true feelings regarding my job” (1 = *strongly disagree* to 7 = *strongly agree*; *α* = 0.80).

#### Power Distance Orientation

Employees rated their own individual power distance orientation with a six-item measure developed by Dorfman and Howell (1988). A sample item included “In most situations, managers should make decisions without consulting their subordinates” (1 = *strongly disagree* to 7 = *strongly agree*; *α* = 0.82).

#### Voice Behavior

Employee voice was self-rated with the 10-item scale developed by Liang et al. (2012), which contains two subscales of promotive voice and prohibitive voice (five items each). In terms of promotive voice, sample items included “The employee raises suggestions to improve the unit’s working procedure” and “The employee makes constructive suggestions to improve the unit’s operation” (1 = *very infrequent* to 7 = *very frequent*; *α* = 0.90).

In terms of prohibitive voice, sample items included “The employee speaks up honestly with problems that might cause serious loss to the work unit, even when/though dissenting opinions exist” and “The employee dares to voice out opinions on things that might affect efficiency in the work unit, even if that would embarrass others” (1 = *very infrequent* to 7 = *very frequent*; *α* = 0.87).

#### Control Variables

Several employees’ demographic variables were included as control variables. We controlled for employees’ gender (0 = *female*, 1 = *male*); age (1 = *under 20 years old*, 2 = *21–25 years old*, 3 = *26–30 years old*, 4 = *31–35 years old*, 5 = *36–40 years old*, 6 = *41–45 years old*, 7 = *above 46 years old*); education level (1 = *vocational school/technical secondary school*, 2 = *high school*, 3 = *vocational/junior college*, 4 = *undergraduate*, 5 = *graduate*); and organizational tenure (1 = *less than 1 year*, 2 = *2–3 years*, 3 = *4–5 years*, 4 = *6–7 years*, 5 = *8–9 years old*, 6 = *10 years or above*) because these demographic variables have been reported to affect individuals’ perceptions of social interactions and their behavioral outcomes (e.g., Ng and Feldman, 2010).

## Results

### Descriptive Statistics and Preliminary Analyses

Table 1 presents the descriptive statistics, including means, standard deviations, correlations, and reliabilities of variables in our models.

**TABLE 1:** Means, standard deviations, correlations, and reliabilities among studied variables.

**Variables**	**Mean**	***SD***	**1**	**2**	**3**	**4**	**5**	**6**	**7**	**8**	**9**	**10**
1. Gender	0.49	0.50	−									
2. Age	3.72	1.24	0.06	−								
3. Education level	3.82	0.67	–0.02	−0.19**	−							
4. Tenure	3.56	1.56	0.02	0.75**	−0.12**	−						
5. Social currency	4.91	1.04	0.00	−0.15**	0.23**	−0.13**	*0*.*91*					
6. Work-related currency	4.56	1.20	0.08	–0.06	0.02	–0.03	0.58**	*0*.*80*				
7. Psychological safety	4.89	1.06	0.06	–0.03	0.22**	–0.07	0.68**	0.42**	*0*.*80*			
8. Power distance orientation	3.75	1.11	0.12**	−0.10*	0.00	−0.17**	0.09*	0.15**	0.22**	*0*.*82*		
9. Promotive voice	4.56	1.16	0.06	–0.05	0.22**	–0.04	0.61**	0.44**	0.65**	0.24**	*0*.*90*	
10. Prohibitive voice	4.32	1.17	0.01	–0.05	0.13**	–0.04	0.55**	0.42**	0.56**	0.30**	0.71**	*0*.*87*

*N = 598. Cronbach’s alphas are presented on the diagonal in italics. SD, standard deviation. Gender: 0 = female; 1 = male. Education: 1 = vocational school, technical secondary school; 2 = high school; 3 = vocational/junior college; 4 = undergraduate; 5 = graduate. Age: 1 = under 20 years old; 2 = 21–25 years old; 3 = 26–30 years old; 4 = 31–35 years old; 5 = 36–40 years old; 6 = 41–45 years old; 7 = above 46 years old. Organizational tenure: 1 = less than 1 year; 2 = 2–3 years; 3 = 4–5 years; 4 = 6–7 years; 5 = 8–9 years old; 6 = 10 years or above. ^∗^p < 0.05, ^∗∗^p < 0.01.*

Overall, we conducted two-step procedure analyses with Mplus 7.4 testing both the measurement model and path analysis separately.

### The Measurement Model and Common Method Variance

In order to ensure construct validity and address potential concern about common method bias, we first conducted confirmatory factor analyses of our constructs before testing our hypotheses. We included all items of the focal six variables. Values of χ*^2^/df* lower than 5, values of comparative fit index (CFI) and Tucker-Lewis index (TLI) higher than 0.90, and values of the root-mean-square error of approximation (RMSEA) lower than 0.08 are regarded as an acceptable fit (Kline, 2010). Table 2 shows that the six-factor model, as we hypothesized, has adequate fit (χ*^2^*/*df* = 2.72, *CFI* = 0.93, *TLI* = 0.93, *SRMR* = 0.05, *RMSEA* = 0.05). This model also indicates a significant improvement comparing to alternative models. Thus, the focal variables are empirically distinct.

**TABLE 2:** Model fit results for confirmatory factor analyses.

**Models**	***χ ^2^***	**Δ *χ ^2^***	***χ ^2/df^***	**SRMR**	**TLI**	**CFI**	**RMSEA**
**SIX-FACTOR MODEL**							
The hypothesized model	1,130.93	–	2.72	0.05	0.93	0.93	0.05
**FIVE-FACTOR MODEL**							
Combing social currency and work-related currency	1,312.41	181.48	3.12	0.05	0.91	0.92	0.06
Combing social currency and psychological safety	1,462.85	331.92	3.47	0.06	0.90	0.91	0.06
Combing promotive voice and prohibitive voice	1,598.47	467.54	3.80	0.05	0.89	0.90	0.07
**FOUR-FACTOR MODEL**							
Combing social currency, work-related currency, and psychological safety	1,631.68	500.75	3.84	0.06	0.88	0.89	0.07
Combing social currency and work-related currency, and combining promotive voice and prohibitive voice	1,776.30	645.37	4.18	0.06	0.88	0.89	0.07
**THREE-FACTOR MODEL**							
Combing social currency, work-related currency, psychological safety, and power distance orientation	2,872.05	1,741.12	6.71	0.09	0.81	0.82	0.10
Combing social currency and work-related currency, combining psychological safety and power distance orientation, and combining promotive voice and prohibitive voice	2,868.13	1,737.2	6.70	0.08	0.81	0.82	0.10
**TWO-FACTOR MODEL**							
Combing social currency, work-related currency, psychological safety, and power distance orientation, and combining promotive voice and prohibitive voice	3,345.81	2,214.88	7.78	0.09	0.78	0.79	0.11
**ONE-FACTOR MODEL**							
Combing all	5,127.12	3,996.19	11.90	0.10	0.70	0.72	0.14

*Δχ^2^ was compared with the hypothesized six-factor model. TLI, Tucker-Lewis index; CFI, comparative fit index; RMSEA, root-mean-square error of approximation; SRMR, standardized root-mean-square residual.*

Furthermore, we addressed the concern of possible common method bias associated with self-reported data by using Harman’s one-factor test (Podsakoff and Organ, 1986). As shown in Table 2, the six-factor model as we hypothesized shows much a better fit than the one-factor model (χ*^2^*/*df* = 11.90, *CFI* = 0.72, *TLI* = 0.70, *SRMR* = 0.10, *RMSEA* = 0.14). Moreover, the explained variance of the first factor from explanatory factor analysis is 37.31%, lower than the bar of 50% (Hair et al., 1998). In addition, we conducted variance inflation factor (VIF) tests, and the values of our variables are all much lower than 10. Thus, multicollinearity is not an issue in our study.

We then conducted path analyses in Mplus 7.4 to test our hypotheses. The proposed model with all the control variables (i.e., gender, age, educational level, and tenure) had a reasonably good fit to the data (χ*^2^*/*df* = 4.83, *CFI* = 0.90, *TLI* = 0.91, *SRMR* = 0.06, *RMSEA* = 0.08). Table 3 shows the results of path analysis of the hypothesized model.

**TABLE 3:** Path analysis results on promotive voice and prohibitive voice.^a^

**Variables**	**Promotive voice**	**Prohibitive voice**
	
	**M1**	**M2**
**CONTROLS**		
Gender	0.01 (0.03)	−0.04 (0.03)
Age	0.00 (0.04)	−0.04 (0.05)
Education level	0.09** (0.03)	0.00 (0.03)
Tenure	0.05 (0.04)	0.08 (0.05)
**PREDICTORS**		
Social currency	0.27*** (0.05)	0.24*** (0.05)
Work-related currency	0.10** (0.04)	0.11** (0.04)
Psychological safety	0.40*** (0.04)	0.37*** (0.04)
Power distance orientation	0.12*** (0.03)	0.20*** (0.03)
**INTERACTIONS**		
Social currency × psychological safety	0.06* (0.03)	0.07* (0.03)
Work-related currency × power distance orientation	0.06* (0.03)	0.01 (0.03)

^*a*^N = 598. Statistics reported are standardized regression coefficients (and standard errors). M = model. *p < 0.05, **p < 0.01, ***p < 0.001.

Hypotheses 1a and 1b posit that social currency is positively related to employee promotive voice (H1a) and employee prohibitive voice (H1b). Table 3 reports our results. It shows that after controlling for an employee’s demographics, employee social currency positively related to both promotive voice [β = 0.27, standard error (*SE*) = 0.05, *p* < 0.001; Model 1] and prohibitive voice (β = 0.24, *SE* = 0.05, *p* < 0.001; Model 2). Thus, Hypothesis 1a and Hypothesis 1b are both supported.

Hypothesis 2a posits that work-related currency is positively related to employee promotive voice while Hypothesis 2b posits that work-related currency is negatively related to prohibitive voice. The results summarized in Model 1 in Table 3 show that employee work-related currency is positively associated with promotive voice (β = 0.10, *SE* = 0.04, *p* < 0.01; Model 1). Thus, Hypothesis 2a is supported. Moreover, as shown in Model 2, the relationship between employee work-related currency and prohibitive voice is positively significant (β = 0.11, *SE* = 0.04, *p* < 0.01) and contrary to our hypothesis. Thus, Hypothesis 2b is not supported.

**FIGURE 2:**
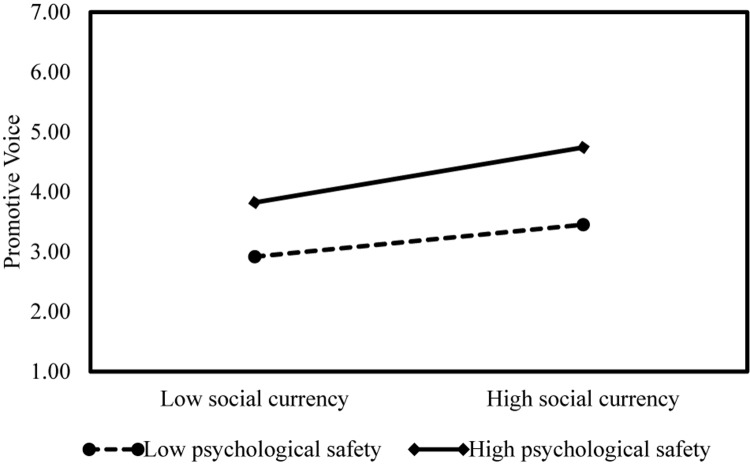
Interactive effects of social currency and psychological safety on promotive voice.

Hypotheses 3a and 3b predict that the positive relationships between social currency and voice are positively moderated by employee psychological safety such that the relationships become stronger when psychological safety is high rather than low. Following Cohen et al. (2003), we centered all continuous variables before creating their product terms. The results from path analysis show that the interaction term of social currency and psychological safety is positively related to employee promotive voice (β = 0.06, *SE* = 0.03, *p* < 0.05; Model 1). In order to further interpret the results, we followed Aiken and West’s (1991) procedures to depict interactions (see Figure 2) and conducted a simple slopes analysis. We conducted hierarchical regression analyses using SPSS 24.0 to obtain the unstandardized outputs. The interaction plot in Figure 2 shows that with low psychological safety (1 *s.d.* below the mean), social currency is significantly related to employee promotive voice (*simple slope* = 0.26, *SE* = 0.06, *p* < 0.001) and weaker, while with high psychological safety (1 *s.d.* above the mean), social currency is significantly related to employee promotive voice (*simple slope* = 0.44, *SE* = 0.09, *p* < 0.001) and stronger. Thus, Hypothesis 3a is supported.

**FIGURE 3:**
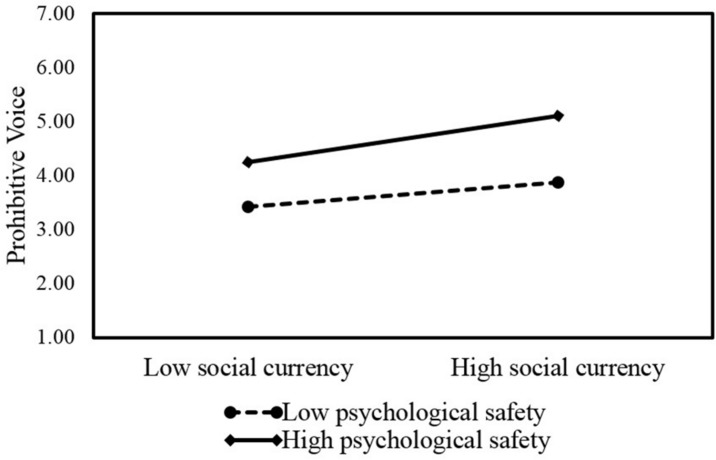
Interactive effects of social currency and psychological safety on prohibitive voice.

Meanwhile, Table 3 also shows that the interaction term of social currency and psychological safety is positively related to employee prohibitive voice (β = 0.07, *SE* = 0.03, *p* < 0.05; Model 2). The interaction plot in Figure 3 also shows a similar pattern such that with low psychological safety (1 *s.d.* below the mean), social currency is significantly related to employee promotive voice (*simple slope* = 0.21, *SE* = 0.06, *p* < 0.01) but weaker, while with high psychological safety (1 *s.d.* above the mean), social currency is significantly related to employee prohibitive voice and stronger (*simple slope* = 0.42, *SE* = 0.09, *p* < 0.001). Therefore, we have strong support for Hypothesis 3b.

**FIGURE 4:**
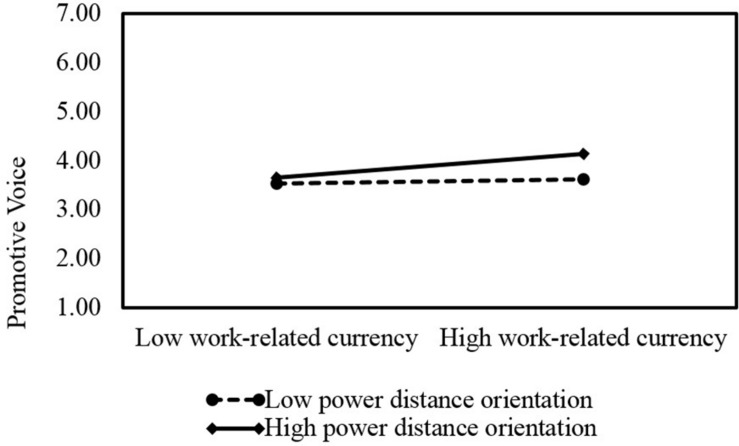
Interactive effects of work-related currency and power distance orientation on promotive voice.

Hypothesis 4a predicts that power distance orientation moderates the relationship between work-related currency and promotive voice such that the relationship becomes stronger when employee power distance orientation is high. Table 3 shows that the interaction term of work-related currency and power distance orientation is positively related to employee promotive voice (β = 0.06, *SE* = 0.03, *p* < 0.05; Model 1). The interaction plot in Figure 4 seems to provide support for our hypothesis such that work-related currency is more strongly related to promotive voice when power distance orientation is high rather than low. Specifically, with high power distance orientation (1 *s.d.* above the mean), work-related currency is positively related to employee promotive voice (*simple slope* = 0.21, *SE* = 0.06, *p* < 0.001); with low power distance orientation (1 *s.d.* below the mean), work-related currency is not positively related to employee promotive voice (*simple slope* = 0.04, *SE* = 0.06, *p* > 0.05). Thus, we have support for Hypothesis 4a.

**FIGURE 5:**
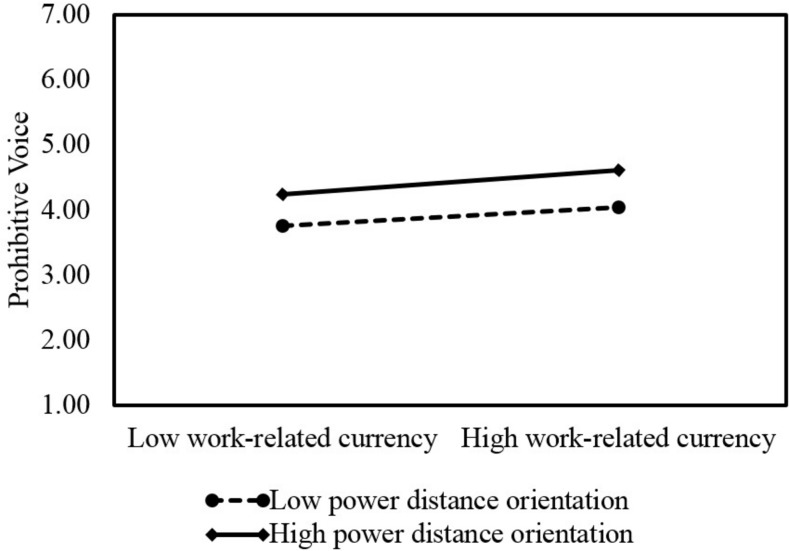
Interactive effects of work-related currency and power distance orientation on prohibitive voice.

Hypothesis 4b predicts that employee power distance orientation moderates the relationship between work-related currency and prohibitive voice such that the hypothesized negative relationship becomes stronger when power distance orientation is high. However, our results do not provide support for this prediction. Table 3 shows that the interaction term of work-related currency and power distance orientation is *not* significantly related to employee prohibitive voice (β = 0.01, *SE* = 0.03, *p* > 0.05; Model 2). We also plotted this interaction in Figure 5. It shows that with high power distance orientation (1 *s.d.* above the mean), work-related currency is positively related to employee prohibitive voice (*simple slope* = 0.16, *SE* = 0.06, *p* < 0.01), but with low power distance orientation (1 *s.d.* below the mean), work-related currency is also significantly related to employee prohibitive voice (*simple slope* = 0.12, *SE* = 0.06, *p* < 0.05). Overall, we do not find support for Hypothesis 4b.

## Discussion

One of the primary goals of this study was to explore how and when employees engage in promotive and prohibitive voice. We drew from social exchange theory and the multidimensional perspective of LMX and focused on the role of exchange relationships between employees and their leaders and the different contents in these exchange relationships. In doing so, we examined the influence of social and work-related currencies on promotive and prohibitive voice, and the moderating effects of psychological safety and power distance orientation.

First, our findings show that both social currency and work-related currency have a significant and positive impact on promotive voice. Contrary to our prediction, results show that work-related currency is positively related to prohibitive voice. We speculate that this positive relationship between work-related currency and prohibitive voice might be explained by the different motives driving voice behavior. Even though engaging in prohibitive voice could potentially distort the strong impression employees have managed to obtain, it is possible that those with high work-related currency can be driven by other-serving motives rather than their own interests to do so. This is consistent with the notion that there are different motives of voice, and scholars should continue to explore the various factors that promote employees’ voice behavior of different kinds (Chamberlin et al., 2017).

Furthermore, our study indicates that the conditions under which promotive voice and prohibitive voice can be explained by exchange relationships vary across different contexts. Specifically, employee psychological safety strengthens the influence of social currency on both types of voice behavior, while employee power distance orientation could only amplify the relationship between work-related currency and promotive voice. It should be pointed out that we do not find strong support for the prediction that employees’ willingness to engage in prohibitive voice is further reduced when power distance orientation is high. From a motives point of view, this, perhaps, can be explained by the possibility that other-serving motives overshadow the influence of self-serving concerns. Nonetheless, our overall finding indicates that the differences in terms of the contents of exchange relationships in different kinds of voice are even more pronounced while considering an individual’s dispositional characteristics. Thus, it contributes to both LMX research and voice research by shedding new light on the underlying mechanisms regarding how employee voice behavior can be explained by employees’ dispositional factors in conjunction with relational factors. In section that follows, we discuss the contributions of our study in detail.

### Theoretical Implications

This study explores the impact of social and work-related currencies on employee promotive and prohibitive voice behavior and the conditions under which the impacts of different currencies of exchange on the two kinds of voice behavior become stronger. Synthesizing extant literature on voice and the multidimensional perspective within LMX research, our study provides the following theoretical implications.

First, we adopted the multidimensional view of LMX in predicting employee voice behavior. Whereas work-related currency stands for the interactions on job-related issues, social currency stands for the interactions on non-job-related issues (Bhal and Ansari, 2007). Adopting the multidimensional view of LMX allows us to further uncover the complex relationship between LMX and employee voice behavior by probing into the role of different contents of the exchange relationship embedded in LMX. We also distinguish between promotive and prohibitive voice and explore their antecedents in a more fine-grained manner. It enables us to unpack the distinct mechanisms through which the different aspects of exchange influence promotive and prohibitive voice.

Furthermore, we theorized the different patterns of interactions between individual factors and relational factors in predicting the two kinds of voice behavior. We examined the moderating roles of psychological safety and power distance orientation, respectively, in the relationships between social and work-related currencies and promotive and prohibitive voice. Our results indicate that both kinds of employee voice behavior are influenced by individual characteristics in conjunction with relational factors but in distinct ways. Specifically, our findings show that psychological safety can further strengthen the relationship between social currency and both kinds of voice, while employee power distance orientation could amplify the relationship between work-related currency and promotive voice. This provides new insights to the literature on how to foster employee voice behavior by incorporating both relational and individual factors. It also reiterates the importance of examining the nature of different workplace interactions and the situational context in which parties interact with each other in this inquiry.

### Practical Implications

Our study has multiple implications for managerial practices. First, prior studies have mostly focused on promotive voice, which emphasizes achieving a better state for the organization (Morrison, 2011). By examining antecedents of both promotive and prohibitive voice, we highlight the importance of prohibitive voice within organizations. Prohibitive voice should attract greater managerial attention in that it can help organizations to avoid harmful things from happening.

Second, encouraging employees to share their ideas or to express their concerns can have critical implications. Leaders play important roles in this regard. They can promote voice behavior by developing and maintaining high-quality exchange relationships with their subordinates. As such, they should be open to communicating with employees and proactively seek input and feedback from employees. They should try to find more ways to encourage prohibitive voice behavior as well.

Third, our results show that the effects of currencies on voice are unequal for people with different psychological safety and power distance orientation. Comparing to power distance orientation, employees’ psychological safety can have more influence in facilitating promotive and prohibitive voice. Leaders should cultivate a harmonious workplace environment within which employees feel psychologically safe. In addition, leaders can further encourage promotive voice behavior by promoting more work-related currencies with employees with high power distance orientation.

### Limitations and Directions for Future Research

Although our study makes several contributions to theory and practice, it is not without limitations. First, we collected data from a single source (the employees). This might lead to common method bias. As such, we followed prior studies and conducted several tests to ensure that common method bias was not an issue in our study. Nevertheless, future studies can be further complemented by data collection from different sources, such as self-reported currencies and individual factors combined with leader-rated voice behavior. Second, our research design was cross-sectional, which serves as an insufficient basis to infer a causal relationship. Future research can benefit from multi-wave longitudinal studies to gain additional insights. Third, we could not draw conclusions about the differential predicting power of the two currencies on the two types of voice. Additional insights might better explain why work-related currency was not significantly related to prohibitive voice in our study. Fourth, our measurement for work-related currency only contained two items. Although Cronbach’s α reached the level of acceptance, in order to obtain robust results, we encourage further research to utilize other measurements to test work-related currency. Finally, we only examined the moderating effects of two important individual factors, psychological safety and power distance orientation, on the relationship between currencies and voice. We believe that research in this vein can benefit from more exploration of different potential moderators in explaining the relationship between currencies and the different kinds of voice.

## Conclusion

In a changing business world, voice can help an organization to achieve and sustain a competitive advantage (Detert and Edmondson, 2011; Whiting et al., 2012). As such, one important question that leaders are increasingly facing today is how they can improve employee promotive and prohibitive voice behavior. This study provides helpful insights. Specifically, we drew from both relational and individual perspectives and examined how social and work-related currencies of exchange can lead to more promotive voice and prohibitive voice. We also explored their different boundary conditions. We contribute to the LMX literature and voice research by being the first to adopt the multidimensional approach to explain the relationship between LMX and promotive and prohibitive voice while taking into account individual dispositional characteristics. We hope our study can encourage more research in this vein to further explore why and when currencies of exchange can influence promotive and prohibitive voice behavior in various contexts.

## Data Availability Statement

The datasets generated for this study are available on request to the corresponding author.

## Ethics Statement

This study was carried out in accordance with the recommendations of the ethics committee of Tsinghua University with written informed consent from all subjects. All subjects gave written informed consent in accordance with the Declaration of Helsinki. The protocol was approved by the ethics committee of Tsinghua University.

## Author Contributions

QZ contacted the company, supervised the data collection, and completed the statistical analysis. DH made contributions in data collecting and manuscript drafting. FW reviewed the literature, and prepared and formatted the manuscript in accordance with Frontiers guidelines.

## Conflict of Interest

The authors declare that the research was conducted in the absence of any commercial or financial relationships that could be construed as a potential conflict of interest.
